# A case of calciphylaxis with an unfavorable outcome^☆^^☆☆^

**DOI:** 10.1016/j.abd.2020.02.012

**Published:** 2020-07-15

**Authors:** Maria Carolina Ribeiro Braga, Susana Strougo, Enoï Guedes Vilar, Sandra Maria Barbosa Durães

**Affiliations:** a Department of Dermatology, Universidade Federal Fluminense, Niterói, RJ, Brazil; b Department of Dermatopathology, Universidade Federal Fluminense, Niterói, RJ, Brazil

Dear Editor,

Calciphylaxis or calcifying uremic arteriolopathy is a rare and serious complication secondary to late-stage chronic kidney disease (CKD).^1^ It is observed in 1% to 4.5% of dialysis patients, especially those undergoing hemodialysis and women.^2^ Hyperparathyroidism (HPT) secondary to nephropathy leads to changes in the metabolism of calcium (Ca) and phosphorus (P), representing the main etiological factor.

The authors report the case of a female patient, aged 50 years, with a history of painful skin lesion with ten days of evolution (Fig. 1), soon followed by a necrotic ulcer in the lower limbs. The patient had CKD secondary to diabetes mellitus and arterial hypertension and had undergone peritoneal dialysis for five years. Dermatological examination showed irregular, erythematous-violaceous plaques, with central necrotic ulcers, measuring from 5 to 10 cm, in the distal third of the lower limbs (Fig. 2). Laboratory investigation revealed an increase in P, Ca, parathyroid hormone (PTH), and alkaline phosphatase, as well as anemia and hypoalbuminemia. Histological examination of the edge of an ulcer indicated a slight superficial perivascular inflammatory infiltrate and foci of Ca deposition in the subcutaneous tissue and vascular wall, confirmed by Von Kossa staining (Fig. 3). Leg radiography revealed vascular calcifications in the topography of the popliteal and posterior tibial arteries, in addition to a slight diffuse increase in soft tissue density. The diagnosis of calciphylaxis was made, based on clinical, radiological, and histopathological findings. Treatment was started with a hypophosphatemic diet and adjustments in the dialysis to correct Ca, P, and PTH, analgesia, and dressing with 1% chloramphenicol on the ulcers. After failure of clinical treatment, surgical debridement and antibiotic therapy with amoxicillin and potassium clavulanate were performed. Due to poor response, the antibiotic drugs were replaced with vancomycin hydrochloride. Despite this conduct, an increase in the necrotic area was observed, and the patient’s clinical condition deteriorated, leading to death due to septic shock.

**Figure 1:**
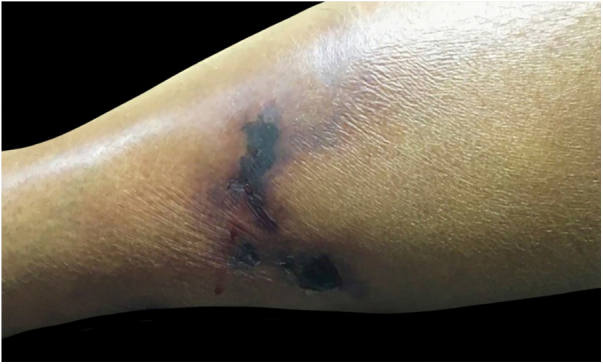
Irregular erythematous-violaceous plaques, with necrotic centers, located in the distal third of the lower limbs.

**Figure 2:**
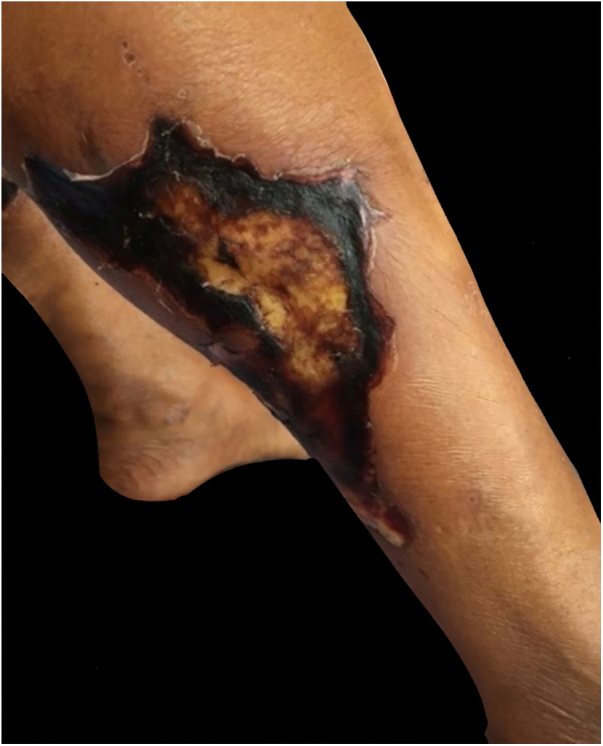
Evolution of the initial lesions to deep ulcerations with irregular and necrotic edges.

**Figure 3:**
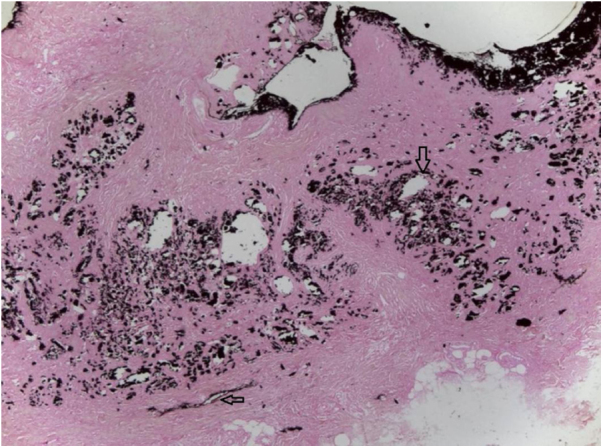
Positive Von Kossa stain on the material deposited in the dermis and blood vessel wall (arrows; ×100).

Calciphylaxis is associated with changes in the metabolism of Ca and P due to HPT secondary to CKD.^3^ In this context, deficits in calcitriol and abnormalities of Ca and vitamin D receptors favor PTH hypersecretion and disease progression. Risk factors include obesity, diabetes, liver disease, use of systemic corticosteroids, and a result of Ca × P above 70 mg/dL.^1^

Very few cases have been described in patients without renal dysfunction; malignant neoplasia, severe liver disease, inflammatory bowel disease, and primary HPT should be ruled out.^4^

Its etiopathogenesis is not fully established and there is calcification and obstruction of the small and medium caliber blood vessels of the dermis, intima and media hyperplasia, and septal and/or lobular subcutaneous necrosis leading to distal ischemia and necrotic ulcers.^2^ Systemic ischemic impairment, such as acute myocardial infarction, stroke, mesenteric ischemia, and peripheral arterial obstructions, may also occur.^4^

Skin lesions are described as painful erythematous-violaceous macules similar to livedo reticularis, progressing to erythematous subcutaneous plaques or nodules with violaceus periphery, which may evolve to central necrosis and ulceration, with no tendency to spontaneous healing and a predilection for the lower limbs.^4^ Sites such as the breasts, buttocks, and abdomen can be affected, with a worse prognosis.^5^

The diagnosis is made on a clinical and laboratory basis, and can be confirmed by histopathology. Laboratory findings include increased PTH, Ca, P, alkaline phosphatase, creatinine, Ca × P product, and anemia. Vasculitis, systemic lupus erythematosus, antiphospholipid antibody syndrome, and Henoch-Schönlein purpura should be ruled out.^2^

Normalization of serum Ca, P, and PTH levels is recommended through a hypophosphatemic diet, Ca-free P chelators, calcimimetics, and sodium thiosulfate.^4^ Pain should be controlled with strict analgesia and systemic antibiotic therapy if necessary.^1^ Parathyroidectomy is reserved for severe and refractory HPT.^5^ The local treatment of ulcers is based on occlusive dressings with fibrinolytics and antibiotics, surgical debridement, and even the use of a hyperbaric chamber.^2^

Mortality reaches 80% and sepsis is the main cause of death, as observed in the present case.^3^ Therefore, it is important to emphasize the need for this entity to be known by dermatologists and nephrologists, since recognizing risk factors and employing preventive measures decreases its occurrence; if the disease is not avoided, early treatment is decisive for reducing mortality and a better prognosis.

## Financial support

None declared.

## Authors’ contributions

Maria Carolina Ribeiro Braga: Conception and planning of the study; elaboration and writing of the manuscript; obtaining, analyzing, and interpreting the data; intellectual participation in propaedeutic and/or therapeutic conduct of studied cases; critical review of the literature.

Susana Strougo: Conception and planning of the study; elaboration and writing of the manuscript; critical review of the literature.

Enoï Guedes Vilar: Effective participation in research orientation; intellectual participation in propaedeutic and/or therapeutic conduct of studied cases; critical review of the manuscript.

Sandra Maria Barbosa Durães: Approval of the final version of the manuscript; effective participation in research orientation; intellectual participation in propaedeutic and/or therapeutic conduct of studied cases; critical review of the manuscript.

## Conflicts of interest

None declared.
